# Impact of the Elastane Percentage on the Elastic Properties of Knitted Fabrics under Cyclic Loading

**DOI:** 10.3390/ma15196512

**Published:** 2022-09-20

**Authors:** Tea Jovanović, Željko Penava, Zlatko Vrljičak

**Affiliations:** Department of Textile Design and Management, Faculty of Textile Technology, University of Zagreb, 10000 Zagreb, Croatia

**Keywords:** knitted fabric, cycle load, elastic limit, elastane, hysteresis curve

## Abstract

Elastic knitted fabrics find numerous applications in the industry for compression stockings, sports and leisure wear, swimwear, ballet wear, etc. During its use, knitwear is subjected to dynamic loading due to body movements. The loading and unloading of the knitted fabric affect the size of the elastic region in which unrecovered deformation completely disappears. This paper deals with the influence of the elastane percentage in the knitted fabric on the elastic properties of the knitted fabric under dynamic loading. For this experiment, three types of yarn were used in different combinations: polyamide (PA), wrapped elastane yarn and bare elastane. The mentioned yarns were used to knit three different groups of plated weft-knitted fabrics (two yarns in a knitted fabric row): without elastane, knitted fabric with a percentage of wrapped elastane, and knitted fabric with a percentage of bare elastane. The percentage of elastane ranged between 0% and 43%. First, standard uniaxial tensile tests were performed on knitted fabric samples until breakage under static load. The force–elongation diagrams obtained are used to determine the elastic limit up to which Hook’s law applies. All knitted fabrics were cyclically tested to the elastic limit. From the obtained loading and unloading curves, unrecovered deformation (unrecovered elongation), elastic elongation and hysteresis index were determined and calculated. The results showed that the percentage of elastane significantly affects the size of the elastic region of the knitted fabric and has no effect on the hysteresis index. Therefore, it is necessary to optimize the elastane percentage for different knitted fabric designs to achieve the best dynamic recovery of the knitted fabric and to design a more stretchable knitted garment that fits the body as well as possible.

## 1. Introduction

The main function of many elastic knitted fabrics is to provide stability and wearing comfort, and not to restrict body movement. For example, knitted fabrics for sports and leisure wear should be comfortable, less sweat-absorbent, breathable, lightweight, durable and suitable for all weather conditions and body movements. Such requirements are affected by the climatic changes to which the knitted fabric is exposed, the number of wears, washes and drying processes. All of the above affect their deformation, change in structure and properties, on the basis of which the results of the force drop and hysteresis index are obtained. Knitted fabrics are textile fabrics that have a much higher elongation in the course direction, the so-called transverse direction, than in the wale direction, the so-called longitudinal direction [1]. Increasingly, highly elastic knitted fabrics are used that are stretchable, comfortable and fit easily on the body. They are usually made with two yarns interlooped into a horizontal row of the knitted fabric. The first yarn is a ground yarn, mainly made of natural fibers, and the second yarn is elastane [2]. For most fabrics, it is very difficult to achieve a stretch of 10–50% and avoid deformations due to stress recovery. Therefore, at the beginning of the use of elastic textiles, especially in sportswear, only knitted fabrics were used, and the next innovation was the addition of elastomeric fibers [3].

Elastomeric fibers have been present for about 70 years. With the improvement of production processes, they are used in a wide and diverse field of textiles [4]. Elastane fibers are also known under trade names such as Lycra, Spandex and Dorlastan. They are like rubber and are very stretchy. Elastane fibers contain a polyurethane bond, which means that the structure of elastic fibers is complex [5]. Elastane is used in all areas where unrecovered elasticity is required, e.g., compression stockings, elastic trousers, sportswear and swimwear, corsets and other knitwear. Elastic garments have a wide range of applications in the field of tight sportswear [6]. Multifilament yarns made of polyamide (PA) or polyester (PES) are often used instead of cotton to produce leisure and sportswear. In a sense, all fibers, except the cheapest raw materials, are high-performance fibers. Single cotton yarns have an extension at break of 3 to 8%, while the elastane has an extension at break of 200 to 900%. Force at break and extension at break are two important factors of any yarn. Force at break is the force required to break the yarn. The percentage increase in length at break is referred to as extension at break. The extension of yarn is the percentage elongation of yarn from the initial length. The mentioned percentages of yarn extension to breakage are important because of the compression force of the knitted fabric on the body. By using the harmonized parameters of yarn, density of knitted fabric and knitted structure, the appropriate structure of the knitted fabric which is suitable for a particular construction of the garment and its application is achieved. Different combinations of yarns, knitted structures and knitted fabric densities provide the appropriate elongation values that correspond to the construction of a garment [7,8,9].

Mukhopadhyay [10] interlooped lycra in combination with cotton and analyzed its effect on the elongation and recovery of the knitted fabric. He found that recovery was better, and that elongation and elasticity were greater in cotton knitted fabrics with a combination of lycra.

Plain single jersey fabrics for making summer T-shirts or undershirts have an elongation at break in the course direction of 150 to 250%, and in the wale direction of 50 to 150%. Double-face knitted fabrics are mostly used in making men’s winter underwear and are four times more stretchable in the course direction than in the wale direction. Chain knitted fabrics made on warp knitting machines with elastane yarns are intended for bathing suits and have approximately equal elongation in the course direction and in the wale direction. Each knitted structure basically gives a different structure of the knitted fabric and even tensile properties [11].

One of the methods for producing knitted fabrics with different elongation is to use plated and partially plated knitted structures in various combinations. When making a plated fabric, a course is formed from two yarns. If it is intended to produce a fuller, and thus more solid structure of the knitted fabric, two identical yarns should be used. However, if it is intended to get knitted fabrics of different tensile properties, two yarns of significantly different tensile properties are used, e.g., the ground yarn is cotton or polyamide, and the other, plating yarn, is elastane. Other combinations include knitting with different structures and counts of the ground and elastane yarn. The combination of plated and partially plated structures, different yarns and sinking depths results in a wide range of elastic knitted structures for different applications. This combination is most commonly used in the production of preventive compression stockings, which exert a compression of 10 to 25 hPa on the leg. In other combinations, instead of the plated structure or with the plated structure, a certain tuck-knitted structure with the specified or similar combinations of yarns can be used. Depending on the function of the product and the construction of the machine, a certain combination will be applied [12,13].

Elastic knitwear should follow body movements. The purpose of the knitted fabric is to follow this elongation and to recover after relaxation. The purpose of elastic knitted fabrics is not to hinder the body movement, but to follow it and return to its original shape [14,15]. Knitted fabrics containing elastane provide a high level of comfort and ease of usage because of elastic properties.

The mechanical properties of textile fibres, i.e., the reactions of the fibres to acting forces and deformations, are technically very important properties that ultimately affect the quality of the end product [16]. Kisilak believes that because of the elasticity and high elasticity of the fibres, the deformations of the knitted fabric are only temporary unless the stresses are too high or last too long, resulting in unrecovered or irreversible deformation [17].

In use, the knitwear is subjected to complex and variable dynamic loads in individual loading and unloading cycles that change the shape of the knitwear. Cyclical measurements are used to analyze the behaviour of the knitted fabric in use. Decrease in force and residual deformation of the knitted fabric are two parameters which well describe a change in the knitted fabric structure after cyclic loads [18]. Su and Yang also found in their studies that elastic recovery decreases with an increasing number of cycles [4]. The results show that with an increasing number of repeat cycles, the plasticity of the elastane increases, while the elasticity decreases, and the elastomeric yarn loses its recovery ability.

If the knitted fabric is successively loaded and unloaded, the stress–strain diagram will have the shape of a loop. This phenomenon of the lagging of strain behind stress is called elastic hysteresis. The surface of the hysteresis loop represents the energy spent on unrecovered deformations during a loading cycle. When repeating the cycle, the surface of the loop slowly increases until breakage occurs. The larger the hysteresis area, the greater the energy loss, i.e., the lower the recovery of the knitted fabric. Elastic knitted fabrics have a higher recovery when the energy loss is lower [19]. Liang and Stewart conducted dynamic tests and defined hysteresis as the difference between stretching (loading) and relaxation (unloading) [20]. Different researchers have studied the cyclic loading of textiles and the occurrence of hysteresis [21,22,23,24].

Penava conducted the knitted fabric off-axes tensile test and for the resulting force–elongation curves proposed a process of determining the zones corresponding to elastic deformation, elastic–plastic deformation, and plastic deformation [25].

The aim of this work was to investigate the influence of the elastane percentage in the knitted fabric on the size of the elastic deformation region of the knitted fabric and on the hysteresis index under cyclic loading.

## 2. Theoretical Analysis

**Figure 1:**
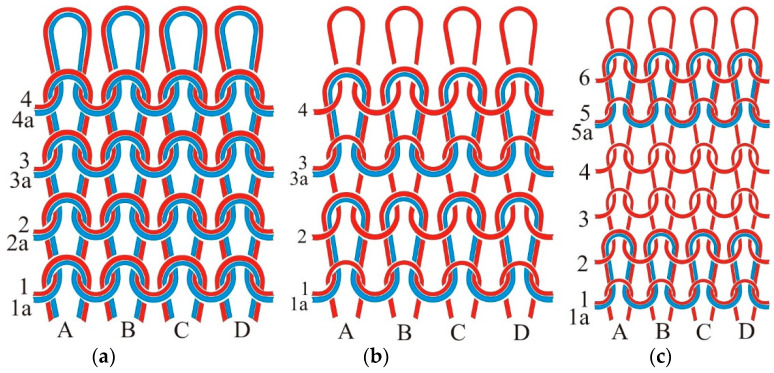
Schematic of plated jersey fabric: (**a**) basic structure; (**b**) partial plating 1 + 1–elastane in every second row of loops; (**c**) partial plating 1 + 3–elastane in every fourth row of loops. 1–6 are basic yarns in course; 1a–4a are plating yarns in course; A–D are wales.

Elastic weft-knitted fabrics (jersey) are usually made of two, three or four yarns interlooped in a horizontal row of loops, where all the yarns form loops and only one is made of elastane. In the basic form of the plated knitted fabric, there are two yarns in a row, one of which is made of elastane (Figure 1a). These knitted fabrics are used for the production of preventive compression stockings, which are mainly worn by pregnant women. In the case of a partially plated knitted fabric, e.g., 1 + 1, the elastane yarn is interlooped into every second row of loops next to the ground yarn. This structure is used in the production of casual trousers, especially for women and children. In the case of partially plated elastic knitted fabric 1 + 3, an elastane yarn is interlooped into every fourth row of loops and the knitted fabric is used to produce elastic fine cotton lingerie containing elastane.

One of the most significant properties of the knitted fabric for clothing is its stretch and elasticity, both in the wale direction and the course direction of stitches. Due to their structure, weft-knitted fabrics are several times more stretchier in the direction of the courses than in the direction of the wales. Knitted fabrics are anisotropic materials whose anisotropy is reflected in different behavior during force action in different directions. During stretching, the loops are deformed in the direction of the forces. Stretching in the course direction changes the shape of the loops, while stretching in the wale direction is achieved exclusively by stretching the yarn.

### 2.1. Load-Elongation Diagram of Knitted Fabric

**Figure 2:**
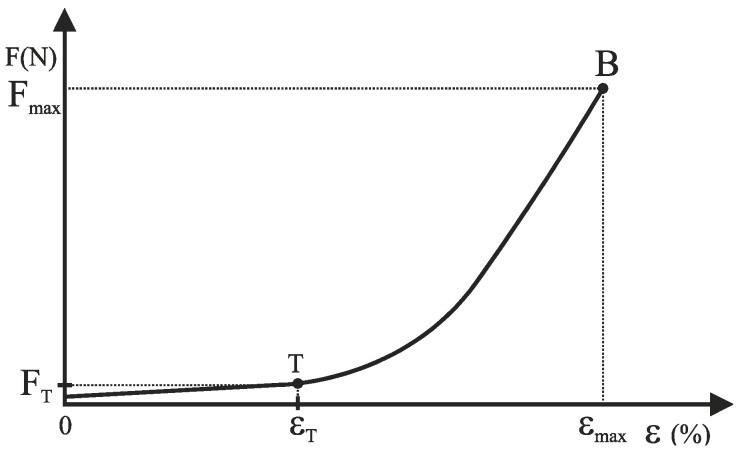
The typical load-elongation (F − ε) diagram of knitted fabric.

Two main directions are distinguished in the structure of the knitted fabric: longitudinal direction (wales) and transverse direction (courses). Under the influence of tensile force F, normal stresses occur in the knitted fabric and its elongation. Figure 2 shows the curve of the ratio of tensile force and elongation of the knitted fabric.

The curve in the tensile force-relative elongation diagram (Figure 2) consists of two parts [25,26]. The first part 0T is a linear region representing the elastic part of the knitted fabric. The second part TB is nonlinear and elastoplastic deformation occurs. Point B represents the maximum force (F_max_) and maximum elongation (ε_max_) of the knitted fabric. In further investigations, the behaviour of the knitted fabric in the elastic region, i.e., up to the point T, is observed [26]. The determination of the point T (elastic limit) is solved by the method of least squares. Tensile force F in the region 0T is expressed as a linear function of the elongation ε [26].

### 2.2. Cycle Force–Elongation Response

**Figure 3:**
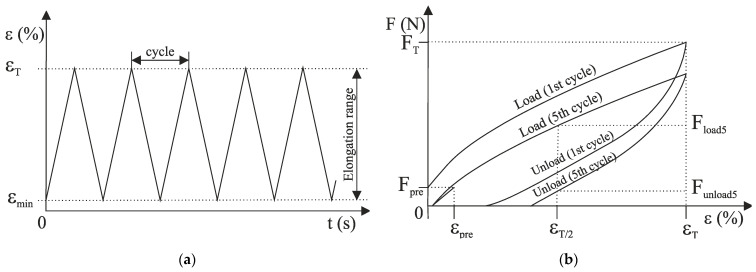
Cyclic testing: (**a**) schematic representation of the change in elongation over time; (**b**) loading–unloading curve.

To observe the behaviour of the knitted fabric in use and to analyse the phenomenon of hysteresis, the knitted fabric should be cyclically loaded [27]. A sample of the knitted fabric is fixed in a tensile tester with two clamps. The upper clamp is fixed, i.e., there is a force-measuring probe on it. The lower clamp is automatically moved down and up by the motor. In this way, the motion is generated for a certain number of cycles, which leads to stresses in the knitted fabric. By moving the lower clamp up and down, the elongation value ε changes, as shown in Figure 3a The elongation values lie in the interval between the minimum elongation value ε_min_ (in our test ε_min_ is zero) and the elongation value ε_T_ at the elastic limit.

In dynamic testing, the lower clamp of the tensile tester moves until it reaches the elongation at a predetermined preload. The sample is then loaded–unloaded five times between two defined elongation points (0 − ε_T_) and reloaded in the next cycle until it reaches the preload value, Figure 3b.

For the 5th cycle, the elastic elongation is calculated according to Equation (1):

(1) $$\varepsilon_{\mathrm{ee}}=\varepsilon_{T}-{\;\varepsilon }_{\mathrm{pre}}$$

ε_ee_ is the elastic elongation, ε_pre_ is the elongation under the action of a preload force at the beginning of the sixth cycle. The hysteresis index is analysed at half the given elongation ε_T_/2 and presented by Equation (2):

(2) $$\mathrm{HYI}=\frac{F_{\mathrm{unload}5}}{F_{\mathrm{load}5}}$$

HYI is the hysteresis index, F_unload5_ is the force read at half the given loading ε_T_/2 in the fifth unloading cycle, F_load5_ is the force read at half the given loading ε_T_/2 in the fifth loading cycle.

For the 5th cycle, unrecovered elongation (ε_ue_) is calculated according to Equation (3):

(3) $$\varepsilon_{\mathrm{ue}}=\varepsilon_{T}-{\;\varepsilon }_{\mathrm{ee}}$$

## 3. Experimental Part

In the experimental part of the work, tensile tests were carried out until the sample of a plated jersey fabric broke under static load. In this test, the values of the tensile forces and the corresponding elongations were determined, and the elastic ranges (elastic limit) were also found. For this purpose, the classical methods, and instruments for testing the tensile properties of knitted fabrics, were used. Dynamic tests of knitted fabric samples, cyclic loading and unloading of knitted fabrics were also carried out. Cycle load-elongation diagrams were obtained.

The experiment was carried out by measuring the deformation of the knitted fabric at the static and dynamic loads, both in the course and wale directions. The aim of this experiment was to determine the influence of the elastane percentage on the elastic range of the knitted fabric, the residual deformation and the hysteresis index.

### 3.1. Materials

#### 3.1.1. Characteristic Parameters of Yarns

Three types of yarn were used for this experiment: polyamide (PA), wrapped elastane yarn and bare elastane. The most important properties of these yarns are listed in Table 1.

**Figure 4:**
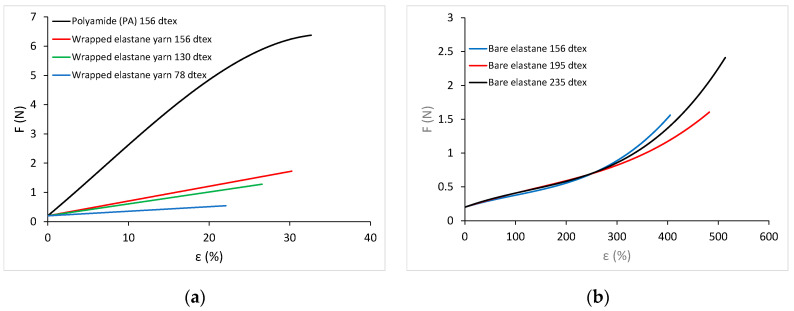
Force–elongation diagrams: (**a**) polyamide yarn and wrapped elastane yarn; (**b**) bare elastane yarn.

The tensile strength properties of the yarn were measured using a Statimat M tensile tester (Textechno H. Stein GmbH & Co. KG, Moenchengladbach, Nordrhein-Westfalen, Germany). The measurements were carried out according to ISO 2062: 2009 and method B. The force–elongation diagram for polyamide and wrapped elastane yarns of different counts is shown in Figure 4a, that for bare elastane of different counts in Figure 4b.

The force–elongation relation for polyamide yarn and for bare elastane yarns is non-linear. For the wrapped elastane yarn, the relation between force and elongation is linear. In the case of bare elastane yarns and of wrapped elastane yarns, the values for breaking forces, breaking elongation and work of rupture increase with increasing yarn counts.

#### 3.1.2. Characteristic Parameters of Knitted Fabrics

Three basic groups of knitted fabric samples were made. In all samples, the course was made from two basically different yarns. Also, in all samples, the ground yarn is polyamide (PA) multifilament with a count of 156 dtex f 68, which is very often used in the production of socks. The first sample (NE) is ground and was made from two equal, abovementioned PA yarns. This sample is used as a reference, and the structures and tensile properties of other samples are compared with it. In the production of the second sample group, in besides the PA multifilament ground yarn with a count of 156 dtex, single-wrapped elastane yarns (elastane core and PA sheath with different PA and elastane percentages) in three different yarn counts were used, which are used in the production of preventive or compression stockings. In the first sample of this group (WE17), a knitted row was made with the abovementioned 156 dtex PA yarn and an elastane yarn, also with a count of 156 dtex-(156/33/10). The second sample of this group (WE16) was made with a 156 dtex PA yarn and a slightly finer elastane yarn, i.e., yarn with a count of 130 dtex-(130/33/20). In the third sample of this group (WE14), a knitted row was made with one of the PA yarns mentioned and an even finer elastane yarn-count 78 dtex-(78/13/7). In the production of these four samples, the tensile force of the PA multifilament ground yarn at the entrance of the knitting zone was 2.5 ± 0.5 cN, and the tensile force of the elastane yarns was 8 ± 2 cN.

The characteristic parameters of the analyzed knitted fabrics are listed in Table 2.

ℓ_PA_—length of interloping the ground (PA) yarn in one loop (mm); ℓ_E_—length of interloping the elastane yarn in one loop (mm); U_PA_—percentage of PA yarn in the knitted fabric (%); U_E_—percentage of the elastane yarn in the knitted fabric (%); NE—non-elastane; WE14—knitted fabric with 14% wrapped elastane; CE32—knitted fabric with 32% bare elastane.

In the third sample group, the abovementioned PA multifilament yarn with a count of 156 dtex as the ground yarn was interloped into one row of the knitted fabric, and bare elastic yarns type 162C (100% elastane) with three different counts were interlooped next to it. The first sample of this group (CE32) with the ground 156 dtex PA yarn in one row also contains an interlooped bare elastane yarn with a count of 156 dtex. In the second sample of this group (CE38), a coarser elastane yarn with a count of 195 dtex was used besides the ground PA yarn, and in the third group (CE43), an even coarser elastane yarn with a count of 235 dtex was used. With these two coarser yarns, i.e., the PA multifilament yarn with a count of 156 dtex and the bare elastane yarn with a count of 235 dtex, practically the highest possible load on knitting needles was achieved for the specified knitting machine gauge. From these three groups of samples or a total of seven basic samples, the structures of knitted fabrics with different elongations were obtained.

The measurement method and the procedure used to examine the thickness of the knitted fabric are specified in the ISO 5084:2003 standard. DIN EN 14971 was used to determine the number of courses and wales of the knitted fabric per unit of length. Standards that were also used to determine the parameters of knitted fabrics:

- ISO 2062:2003: Textiles—Yarn from packages. Determination of single-end breaking force and elongation at break.
- ASTM D8007-15 (2019) was used to determine the wale and course counts of weft-knitted fabrics per unit of length.
- ISO 3801:1977. Determination of mass per unit length and mass per unit area.

### 3.2. Methods

A single bed circular knitting machine with a gauge of E17, needle bed diameter 95 mm (3¾ ″) knitted with 200 needles to make tubular elastic knitted fabrics. The individual samples were 500 to 800 mm long and have a uniform structure that is used in the production of prevention and compression stockings. The circumference of the tubular knitted fabric ranged from 150 to 200 mm. This structure of the knitted fabric is suitable for making the part of the stocking that wraps above the ankle and in the lower part of the leg calf, where the circumference is usually from 200 to 300 mm. Prior to testing, all samples were conditioned under standard atmosphere conditions (relative humidity 65 ± 2 %, at a temperature of 20 ± 2 °C). For this test, standard samples measuring 200 mm × 50 mm were cut and clamped in the clamps of the device at a distance of 100 mm. Five measurements were taken for each knitted fabric sample.

The tensile properties of all knitted fabric samples were tested according to ISO 13934-1:2013 using the test strip method on a Statimat M tensile tester (Textechno H. Stein GmbH & Co. KG, Mönchengladbach, North Rhine-Westphalia, Germany). After determining the elastic limits (point T) for all knitted fabric samples, the cyclic loading of the knitted fabric samples was carried out up to a predetermined elastic limit.

Dynamic tests of knitted fabric samples were performed according to DIN 53835-2-Testing of textiles; determination of the elastic behavior of single and plied elastomeric yarns by repeated application of tensile load between constant extension limits.

## 4. Results and Discussion

**Figure 5:**
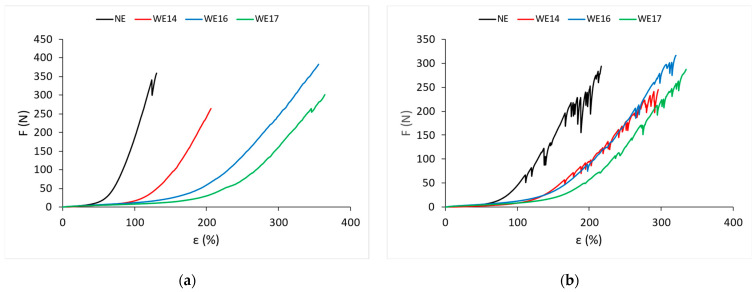
Force–elongation diagrams for knitted fabric samples with polyamide yarn and wrapped elastane yarns: (**a**) wales direction; (**b**) courses direction.

Under the effect of the tensile force F, the corresponding longitudinal deformation, i.e., elongation ε, was measured. The mean values of the measurement results of the effect of the tensile force F and the corresponding elongation ε for the non-elastane knitted fabric samples (NE) and with a different percentage of wrapped elastane (WE) are shown in Figure 5a for the wale direction and in Figure 5b for the course direction.

**Figure 6:**
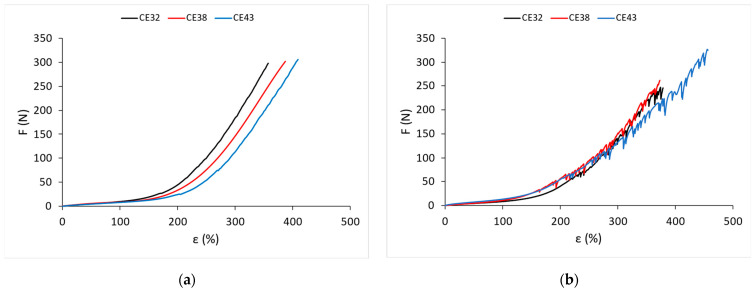
Force–elongation diagrams for knitted fabric samples with bare elastane: (**a**) wales direction; (**b**) courses direction.

The mean values of the measurement results of the effect of the tensile force F and the corresponding elongation ε for the knitted samples with different percentages of bare elastane (CE) are shown in Figure 6a for the wale direction and in Figure 6b for the course direction.

The corresponding mean values of breaking force, elongation at break and work of rupture for the non-elastane knitted fabric samples (NE) and with a different percentage of wrapped elastane (WE) are listed in Table 3, and for the knitted fabric samples with bare elastane (CE), are listed in Table 4. The results presented refer to the wale and course directions of the knitted fabrics.

The knitted fabrics with a percentage of wrapped elastane have significantly higher values of breaking elongation than the non-elastane knitted fabrics. The elongation at break increases with an increasing percentage of wrapped elastane. Elongation at break values increases in the wale and course directions with increasing elastane percentage.

When increasing the percentage of bare elastane in the knitted fabrics, the values of breaking elongation, breaking force, and work of rupture increase.

When knitted fabric samples are stretched with uniaxial tensile force after the linear region, elastoplastic and plastic (unrecovered) deformations occur that are undesirable during the use of knitted fabrics. Up to the elastic limit (point T), the knitted fabric behaves completely elastic. The values of tensile force and corresponding elongation at the point of elasticity are given in Table 5.

Non-elastane knitted fabric (NE) has the lowest elastic limit, namely, in the wale direction amounting to 21.76% and 32.32% in the course direction. The action of tensile forces higher than 2.9 N on such knitted fabrics already leads to the occurrence of elastoplastic and unrecovered deformations. Such knitted fabrics have a small elastic region. In the case of elastane-wrapped knitted fabrics, the elastic limit increases (elongation values and force increase) in relation to non-elastane knitted fabric (NE), so that elastane-wrapped knitted fabrics can withstand higher loads in the elastic range. When the percentage of wrapped elastane increases, the elastic limit rises from 50.8 to 73.4% in the course direction and from 58.24 to 105.24% in the wale direction. The knitted fabric with the highest percentage of bare elastane (CE 43) has the highest elastic limit at which the value of elongation is 119.16%. The elongation at the T-point of knitted fabric CE43 is six times greater in the wale direction than in non-elastane knitted fabric (NE) and almost three times greater in the course direction. The elastic limit (T) increases with the percentage of elastane in the knitted fabric in both the wale direction and in the course direction, whereby this increase is greater in the wale direction. Thus, different percentages of elastane in the knitted fabric can change its elastic properties.

**Figure 7:**
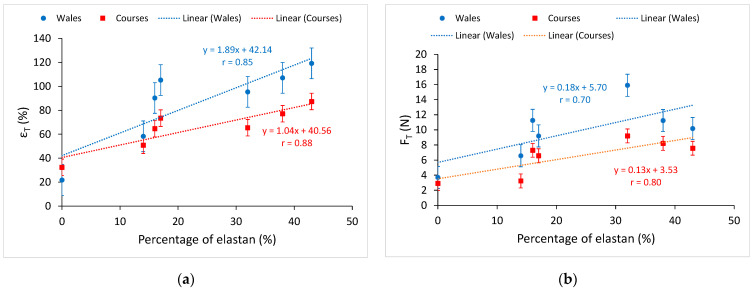
Elastic limit values depending on the percentage of elastane: (**a**) elongation ε_T_ at point T; (**b**) tensile force F_T_ at point T.

The statistical concepts correlation and regression, which are used to evaluate the relationship between two variables, are used in this paper [28,29]. Figure 7a shows the relation between the values of the knitted fabric elongation at the elastic limit ε_T_ and the elastane percentage. This relation is linear both in the wale direction and in the course direction. When the elastane percentage increases, the elongation at the elastic limit also increases linearly. For the wale direction, the correlation coefficient is r = 0.85, and for the course direction, r = 0.88, which is a very high correlation. However, when comparing samples made with elastane yarns wrapped with PA filaments, the scatter of results is greater than when analyzing samples made with bare elastane yarns. These differences are patterned by the structure of the yarn and the different number and yarn count of the filaments that wrap the elastane core of the yarn. By interloping the bare elastane yarn with the PA textured ground yarn, gradually and linearly, without major variation, the elasticity limit of this type of knitted fabric increases.

Figure 7b shows the relation between the values of the tensile force of the knitted fabric at the elastic limit ε_T_ and the elastane percentage. This relation is linear both in the wale direction and in the course direction. For the wales direction, the correlation coefficient is r = 0.70, and for the courses direction, r = 0.80, which is significantly less than the elongation at the elastic limit. Spreads are more irregular in knitted fabric made with elastane wrapped yarns. It is interesting to note that with an increase in the yarn count of the bare elastane yarn, i.e., the proportion of elastane, the tensile force decreases at the end of the elastic deformation of the knitted fabric. This phenomenon can be explained by the higher density of the knitted fabrics and lower porosity.

**Figure 8:**
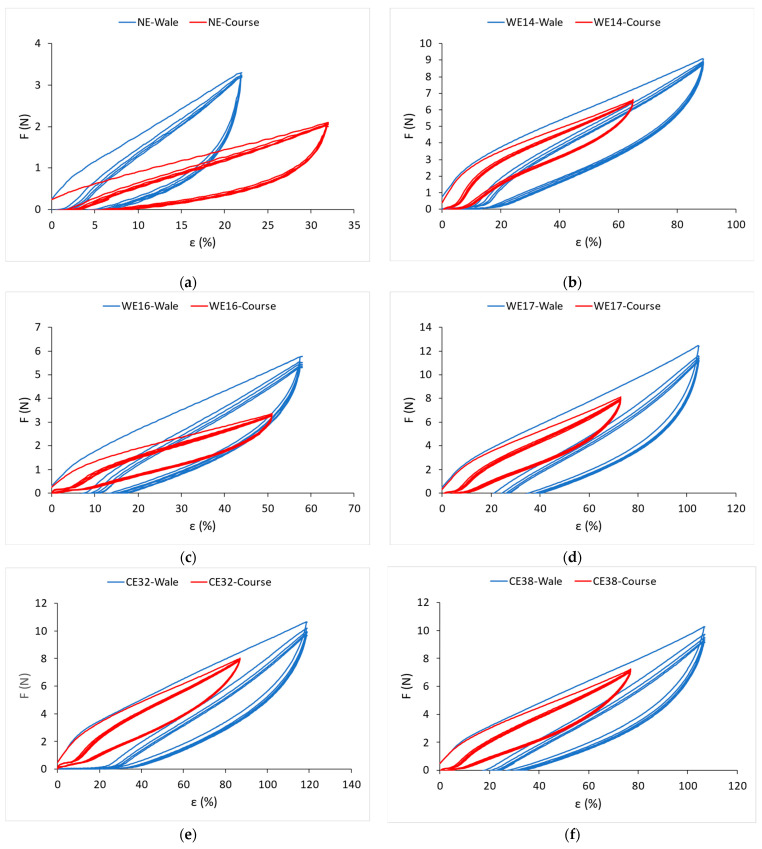

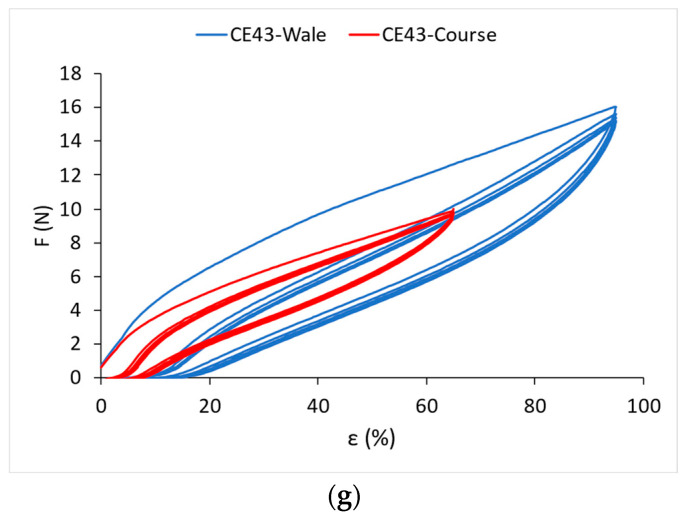
Diagrams of the results of cyclic loading and unloading of the knitted fabrics up to the elastic limit: (**a**) non-elastane knitted fabric; (**b**) knitted fabric with 14% wrapped elastane; (**c**) knitted fabric with 16% wrapped elastane; (**d**) knitted fabric with 17% wrapped elastane; (**e**) knitted fabric with 32% bare elastane; (**f**) knitted fabric with 38% bare elastane; (**g**) knitted fabric with 43% bare elastane.

Figure 8 shows the diagrams of the results of cyclic loading and unloading of the knitted fabrics up to the elastic limit for all tested samples.

The values of unrecovered elongation (ε_ue_), elastic elongation (ε_ee_), and hysteresis index (HYI) in the fifth loading–unloading cycle of the knitted fabrics in the wale direction are listed in Table 6, and for the course direction in Table 7. Equations (1)–(3) were used to calculate the values.

**Figure 9:**
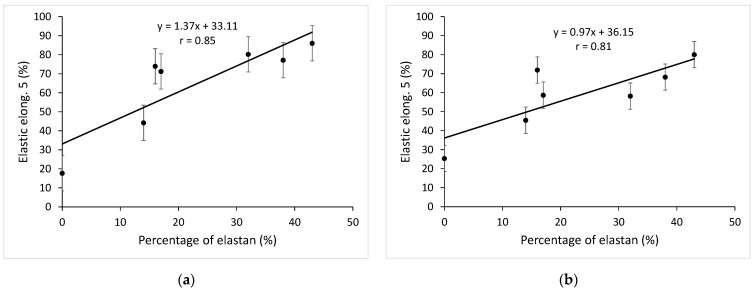
Relation between the percentage of elastane in the knitted fabric and the value of the elastic elongation of the knitted fabric in the fifth cycle of cyclic loading–unloading: (**a**) in the wale direction; (**b**) in the course direction.

The relation between the elastane percentage in the knitted fabric and the value of the elastic elongation of the knitted fabric in the fifth cycle of cyclic loading and unloading in the wale direction is shown in Figure 9a, and in the course direction, in Figure 9b.

This relation is linear both in the wale direction and in the course direction. By increasing the elastane percentage, the elastic elongation also increases linearly in the fifth cycle of cyclic loading. For the wale direction, the correlation coefficient is r = 0.85, and for the course direction, r = 0.81, which is a very high correlation. The elastic elongation in the fifth cycle for the knitted fabric with 43% bare elastane (CE43) is 4.86 times greater than the elastic elongation of the non-elastane knitted fabric (NE) in the wale direction and 3.15 times greater in the course direction. An increase in the percentage of elastane (wrapped and unwrapped) in the knitted fabric causes an increase in the elastic deformation of the knitted fabric even under cyclic loading. As in the previous cases, the analysis results of knitted fabrics made with elastane-wrapped yarns have larger and more irregular scatters than knitted fabrics made with bare elastane yarns. The reason is also in the different structures of elastane yarns.

**Figure 10:**
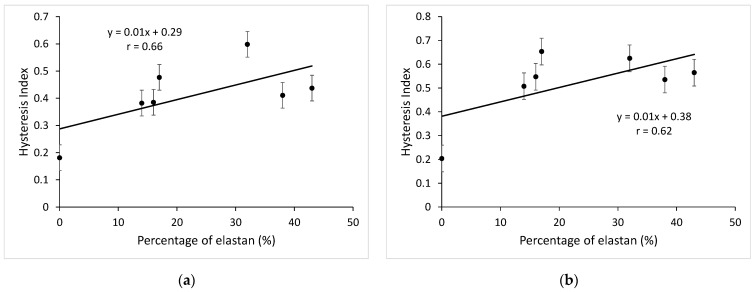
Relation between the percentage of elastane in the knitted fabric and the hysteresis index: (**a**) in the wale direction; (**b**) in the course direction.

Figure 10a shows the relation between the elastane percentage of the knitted fabric and the hysteresis index in the wale direction and Figure 10b in the course direction.

The correlation coefficient is low, and it is not possible to talk about the linear dependence of the elastane percentage in the knitted fabric and the hysteresis index. If only elastane knitted fabrics are analyzed (Table 6 and Table 7), it can be seen that elastane percentage has no effect on the hysteresis index. However, it is interesting to relate the hysteresis index to the yarn count of the interloped elastane yarns. Knitted fabrics with finer wrapped elastane yarns (WE14) and knitted fabrics with coarser bare elastane yarns (CE43) in the course direction give a similar hysteresis index, but in the wale direction the difference between the hysteresis index is greater.

The designed, manufactured and analyzed samples of elastic knitted fabrics with PA and wrapped elastane yarns can be used in the production of preventive compression stockings that are gladly worn by pregnant women or the elderly. When making the legs of these stockings, different lengths of yarn will be woven into the individual courses of knitted fabrics in order to obtain a structure with a different amount of stretching, and thus compression on a particular part of the leg. Knitted fabrics made with PA and bare elastane yarns can be used in making recreational long pants or T-shirts and underwear for mountaineers. When making cuts for this type of clothing, the amount of elastic stretch must be used in order to obtain clothing that fits comfortably against the body.

## 5. Conclusions

In static testing of knitted fabrics, an increase in the elastane percentage in the knitted fabric leads to an increase in the elastic limit of the knitted fabric, i.e., the knitted fabric is stretched to higher elongation values in the elastic region.

Cyclic loading affects the tensile properties of knitted fabrics.

From the test results, it can be concluded that if the occurrence of unrecovered deformation of the knitted fabric under cyclic loading is to be prevented, it is recommendable that the predetermined value to which the knitted fabric stretches (upper elongation limit), Figure 3a, is less than or equal to the value of the elongation at the elastic limit ε_T_.

To reduce the effect of dynamic load on the knitted fabric, it is definitely recommended to use bare, single- or double-wrapped elastane yarn.

The results of cyclic tests have shown that the elastane percentage significantly affects the size of the elastic region of the knitted fabric and has no influence on the hysteresis index. Therefore, it is necessary to optimize the percentage of elastane for different designs of elastic knitted fabrics in order to achieve the best possible dynamic recovery of the knitted fabric and the fit of the garment to the body.

With an increase in the percentage of elastane from 0 to 38%, the elastic area for knitted fabrics increases from 21.76 to 107.03%, the elastic limit is higher and this enables the knitted fabrics to withstand higher loads in the elastic area and return to its original state after unloading (Table 5).

With less yarn interloping in the course of knitted fabric, the stretching of this kind of knitted fabric is also less, and it is used when making compression stockings around the ankle. A higher stretching is obtained with a longer length of yarn interloping in the course of knitted fabric, whereby a higher stretch of the knitted fabric is also achieved, which is needed on the sole of the leg or below the groin.

When making any compression garments, especially preventive compression stockings, it is necessary to match the fineness of the basic and elastane yarns and their structure. When making stockings with less compression, e.g., up to 42 hPa (32 mmHg), the basic PA yarn and elastane yarn can be of the same or similar fineness, e.g., 78, 110 or 156 dtex. However, when making stockings with higher compression, the elastane yarn is much coarser than the base yarn and has a fineness of, for example, 195, 235 or 300 dtex.

## Figures and Tables

**Table 1:** Characteristic parameters of yarns.

Material Type	Polyamide (PA)	Wrapped Elastane Yarn	Wrapped Elastane Yarn	Wrapped Elastane Yarn	Bare Elastane	Bare Elastane	Bare Elastane
Yarn Count (dtex)	156	156	130	78	156	195	235
Breaking Elongation (%)	32.64	30.24	26.56	22.08	405.28	482.40	513.92
Breaking Force (N)	6.38	1.73	1.28	0.55	1.56	1.60	2.41
Tenacity (cN/tex)	40.87	11.07	9.84	6.99	10.01	8.22	10.26
Work of Rupture (N·mm)	124.90	29.13	19.64	8.27	270.82	361.99	469.38

**Table 2:** Test results for plaited knitted fabric parameters.

Fabric Code	Fabric Type	ℓ_PA_ (mm)	ℓ_E_ (mm)	U_PA_ (%)	U_E_ (%)	Knitted Fabric Spirality (°)	Thickness (mm)	Mass per Unit Area (g/m^2^)
**Non-elastane knitted fabric**								
NE	Plaited	3.93	0	100	0	0	1.09	200
**Knitted fabrics with a percentage of wrapped elastane**								
WE14	Plaited	3.76	2.46	86	14	2	1.13	225
WE16	Plaited	3.65	1.56	84	16	3	1.18	260
WE17	Plaited	3.81	1.89	83	17	5	1.23	280
**Knitted fabrics with a percentage of bare elastane**								
CE32	Plaited	3.86	2.39	68	32	3	1.21	310
CE38	Plaited	3.88	2.65	62	38	1	1.17	320
CE43	Plaited	3.81	2.01	57	43	5	1.27	380

**Table 3:** Mean values of elongation at break, breaking force, and work of rupture for the non-elastane knitted fabrics and with a percentage of wrapped elastane.

	Wales				Courses			
	NE	WE14	WE16	WE17	NE	WE14	WE16	WE17
Breaking Elongation (%)	130.4	206.6	356.3	364.5	217.3	295.7	320.3	334.4
Breaking Force (N)	359.5	263.6	383.5	302.0	293.8	244.7	316.9	287.8
Work of Rupture (kN·mm)	12.55	12.44	35.33	25.25	19.71	20.62	28.45	24.60

**Table 4:** Mean values of elongation at break, breaking force, and work of rupture for the knitted fabrics with bare elastane percentage.

	Wales			Courses		
	CE32	CE38	CE43	CE32	CE38	CE43
Breaking Elongation (%)	358.1	387.8	409.3	379.0	373.4	456.6
Breaking Force (N)	297.3	301.4	305.3	247.4	261.8	326.8
Work of Rupture (kN·mm)	27.11	29.94	30.36	26.07	27.49	46.72

**Table 5:** Elongation values and corresponding force (ε_T_, F_T_) at the elastic limit for all knitted fabric samples.

	Wale Direction		Course Direction	
Fabric	ε_T_ (%)	F_T_ (N)	ε_T_ (%)	F_T_ (N)
NE	21.76	3.70	32.32	2.90
WE14	58.24	6.58	50.80	3.23
WE16	90.22	11.26	64.60	7.29
WE17	105.24	9.20	73.40	6.57
CE32	95.32	15.91	65.40	9.20
CE38	107.03	11.23	77.08	8.22
CE43	119.16	10.17	87.32	7.57

**Table 6:** Unrecovered elongation (ε_ue_), elastic elongation (ε_ee_), hysteresis index (HYI) in the 5th cycle in the wale direction.

Fabric	NE	WE14	WE16	WE17	CE32	CE38	CE43
ε_ue_ (%)	4.3	13.8	31.1	17.8	14.8	29.9	33.0
ε_ee_ (%)	17.7	44.2	73.9	71.2	80.2	77.1	86.0
HYI	0.181	0.382	0.385	0.477	0.598	0.411	0.437

**Table 7:** Unrecovered elongation (ε_ue_), elastic elongation (ε_ee_), hysteresis index (HYI) in the 5th cycle in the course direction.

Fabric	NE	WE14	WE16	WE17	CE32	CE38	CE43
ε_ue_ (%)	6.6	5.5	1.1	6.3	6.8	8.8	7.0
ε_ee_ (%)	25.4	45.5	71.9	58.7	58.2	68.2	80.0
HYI	0.204	0.507	0.547	0.653	0.625	0.536	0.565
